# Potential for treatment benefit of STING agonists plus immune checkpoint inhibitors in oral squamous cell carcinoma

**DOI:** 10.1186/s12903-021-01813-8

**Published:** 2021-10-08

**Authors:** Chao Zhu, Jiang Li, Mianfeng Yao, Changyun Fang

**Affiliations:** Department of Stomatology, Xiangya Hospital, Central South University, Changsha, China

**Keywords:** cGAS-STING pathway, Immune microenvironment, Oral squamous cell carcinoma, Immune checkpoint inhibitors

## Abstract

**Background:**

DNA-sensing receptor cyclic GMP–AMP synthase (cGAS) and its downstream signaling effector stimulator of interferon genes (STING) present a novel role in anti-tumor immunity. Recently, the combination of cGAS-STING agonists and immunotherapy achieved promising results in some tumor types. The correlation between cGAS-STING signaling pathway and the tumor immune microenvironment in patients with oral squamous cell carcinoma (OSCC) is unclear.

**Methods:**

We utilized RNA sequencing and clinical data of OSCC patients from the TCGA database to investigate the correlation between cGAS-STING signaling pathway and the tumor immune microenvironment. Six cGAS-STING related genes were obtained from previous studies to establish the enrichment score of cGAS-STING pathway. The differences in survival rate, immune cell infiltration, immune-related genes expression and immune-related biological pathways were studied in the cGAS-STING clusters.

**Results:**

We observed a better prognosis of OSCC patients in the cGAS-STING high cluster. The infiltration ratio of immune cells and the expression profiles of immune-related genes were elevated when the cGAS-STING pathway is activated. The differentially expressed genes between high and low cGAS-STING clusters were enriched in immune-related biological pathways.

**Conclusions:**

Our findings suggest the potential benefit of combining STING agonists and immune checkpoint inhibitors in OSCC patients.

**Supplementary Information:**

The online version contains supplementary material available at 10.1186/s12903-021-01813-8.

## Background

Oral squamous cell carcinoma (OSCC) is one of the common tumors in head and neck region. Despite the advanced management of tumors including surgical resection, with or without radiotherapy or chemoradiotherapy, the survival rate is around 50% [1]. Recent studies on cancer immunotherapy, especially immune checkpoint inhibitors (ICI), have brought significant survival improvements [2]. With the success of immunotherapy, nivolumab and pembrolizumab have both been approved for the treatment of recurrent or metastatic head and neck squamous cell carcinoma (HNSCC). The challenge is that only a fraction of HNSCC patients respond to ICI [3, 4]. The limitations of ICI highlight the need to develop combinatorial approaches that may enhance the efficacy of ICI.

cGAS-STING pathway is a major component of innate immune system. cGAS is a cytosolic double-stranded DNA (dsDNA) sensor that catalyzes the production of cyclic GMP-AMP (cGAMP), which binds to and activates STING [5]. Recent studies have revealed a novel role of the cGAS-STING pathway in cancer development and its potential as a therapeutic target. The activation of cGAS-STING pathway boosts anti-tumor immunity by inducing type I interferon production, which in turn promotes dendritic cell (DC) priming and T cell priming in the tumor microenvironment (TME) [6–8]. However, STING activation, in turn initiates immunosuppressive molecules, such as PD-L1, to prevent tumor clearance [9]. Thus, the combination of STING agonists and ICI has the possibility to overcome the barriers and improve the therapeutic effects of ICI. Promising benefit has been achieved by administration STING agonist in combination with ICI [10, 11].

Therefore, we investigated the correlation between cGAS-STING signaling pathway and the TME, and its association with the survival rate of OSCC patients using data obtained from The Cancer Genome Atlas (TCGA) database. Our results suggest that cGAS-STING pathway targeted therapy with ICI may hold great therapeutic promise for the treatment of OSCC.

## Methods

### Data sources

RNA sequencing and clinical data of 334 OSCC patients were obtained from TCGA data portal (https://portal.gdc.cancer.gov). 7 cases were excluded due to missing follow-up information and overall survival (OS) time less than one month. Finally, 327 OSCC cases were included in this study.

### Analysis of cGAS-STING clusters and immune landscape

The enrichment score of the cGAS-STING pathway was calculated using ssGSEA (single-sample gene set enrichment analysis) method based on previously published six key molecules (cGAS, STING1, TBK1, IRF3, CCL5 and CXCL10) [12, 13]. Data on five immune expression signatures, stromal fraction, and leukocyte fraction was obtained from a previously published study from the TCGA group [14]. The gene set representing 28 immune cell subpopulations was used to quantify the infiltration ratio of immune cells [15]. The proportion of immune cell infiltration was estimated by the ssGSEA method in the Gene Set Variation Analysis (GSVA) R package and visualized by heatmap R package [5, 16]. The immune and estimate scores were downloaded from ESTIMATE database [17].

### The expression profiles of immune-related genes

The gene set of 75 immune markers related to the immune response in the tumor microenvironment was obtained from a previous study [14].

### Identification of differentially expressed genes and functional enrichment analysis

To identify differentially expressed genes (DEGs) between the cGAS/STING pathway clusters, the limma R package was used with cutoff of |log_2_FC|≥ 1.0 and false discovery rate (FDR) < 0.05 [18]. Gene Ontology (GO) terms enrichment analysis of the DEGs was carried out using the Metascape [19] and visualized by ggplot2 R package [20].

### Statistical analysis

Data comparison between cGAS/STING pathway clusters was performed via two-tailed t test and multiple t tests with FDR < 0.05 for continuous comparisons. The correlation between cGAS/STING scores and immune cell scores was determined by Pearson correlation test. Correlation matrix of the ratio of 28 immune cells in the TME and correlation matrix of the expression of immune signatures were calculated and visualized using the corrplot R package [21]. Positive correlations were displayed in blue and negative correlations in red color. Overall survival (OS), disease-specific survival (DSS) and progress free survival (PFS) were plotted using Kaplan–Meier curves and calculated using the Cox regression analysis. In all analyses, a *P* value of a two-tailed test less than 0.05 was thought to be statistically significant. All statistical analyses were conducted by GraphPad Prism v8.0.2 and R software v4.0.5.

## Results

### Correlation between cGAS/STING clusters and the 10-year survival rate of OSCC patients

**Fig. 1:**
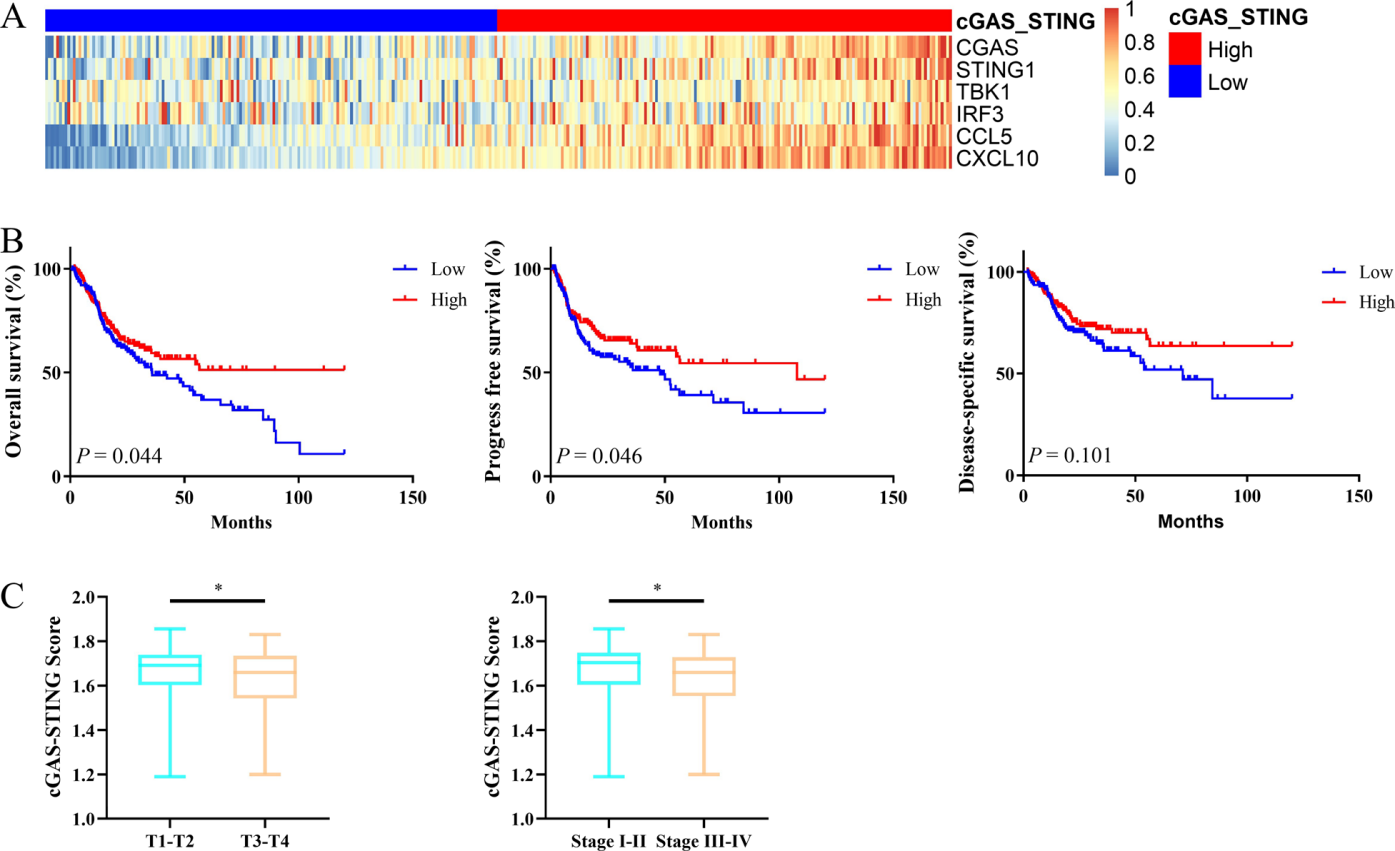
cGAS-STING cluster analysis of the prognosis of OSCC patients. **a** Heatmap of the expression of cGAS-STING related genes in different clusters. **b** The correlation between cGAS-STING clusters and 10-year survival rate of OSCC patients. **c** The distribution of cGAS-STING score in T stage and clinical stage of OSCC patients. All *P* values for significance (< 0.05) represent comparisons via two-tailed t test and Cox regression analysis. *P value < 0.05, **P value < 0.01, ***P value < 0.001, and ****P value < 0.0001

We calculated the enrichment score of the cGAS/STING pathway based on the expression values of six key molecules (Fig. 1a). OSCC patients were divided into two clusters with median value of the enrichment score: the cGAS/STING high score cluster had scores above the median value (n = 164) and the cGAS/STING low score cluster under the median value (n = 163). Patients with high cGAS-STING score showed longer OS and PFS, the association for DSS was borderline significant (Fig. 1b). The cGAS-STING score was relatively lower in T3-T4 group and clinical stage III-IV group (Fig. 1c).

### The immune landscape of cGAS/STING clusters

**Fig. 2:**
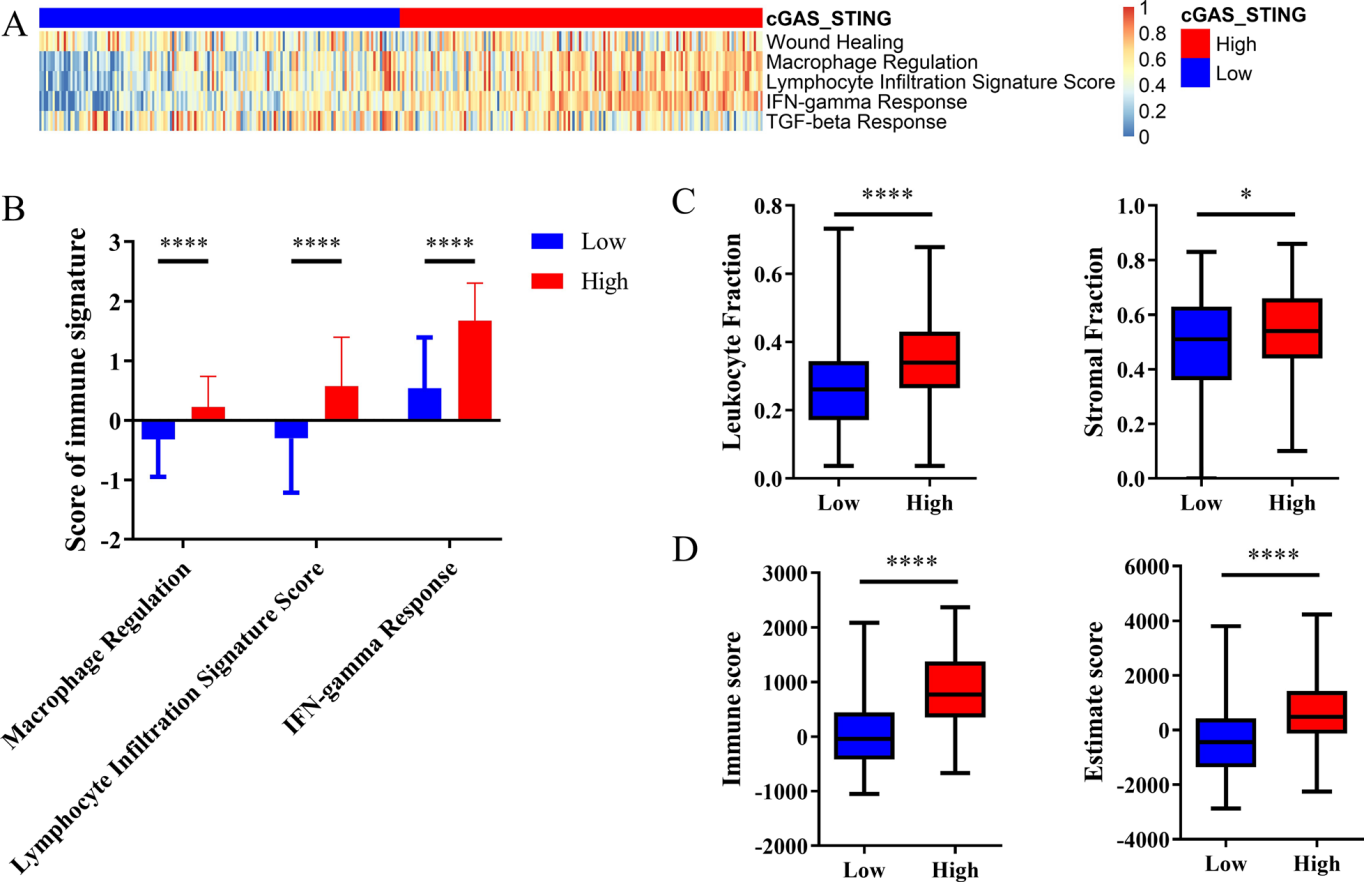
Immune patterns of the cGAS-STING clusters. **a, b** Five representative immune signatures in the cGAS-STING clusters. **c** Stroma fraction and leukocyte fraction of the two cGAS-STING clusters. **d** Immune score and Estimate score of the two cGAS-STING clusters. All *P* values for significance (< 0.05) represent comparisons via two-tailed t test and multiple t tests with FDR < 0.05. *P value < 0.05, **P value < 0.01, ***P value < 0.001, and ****P value < 0.0001

**Fig. 3:**
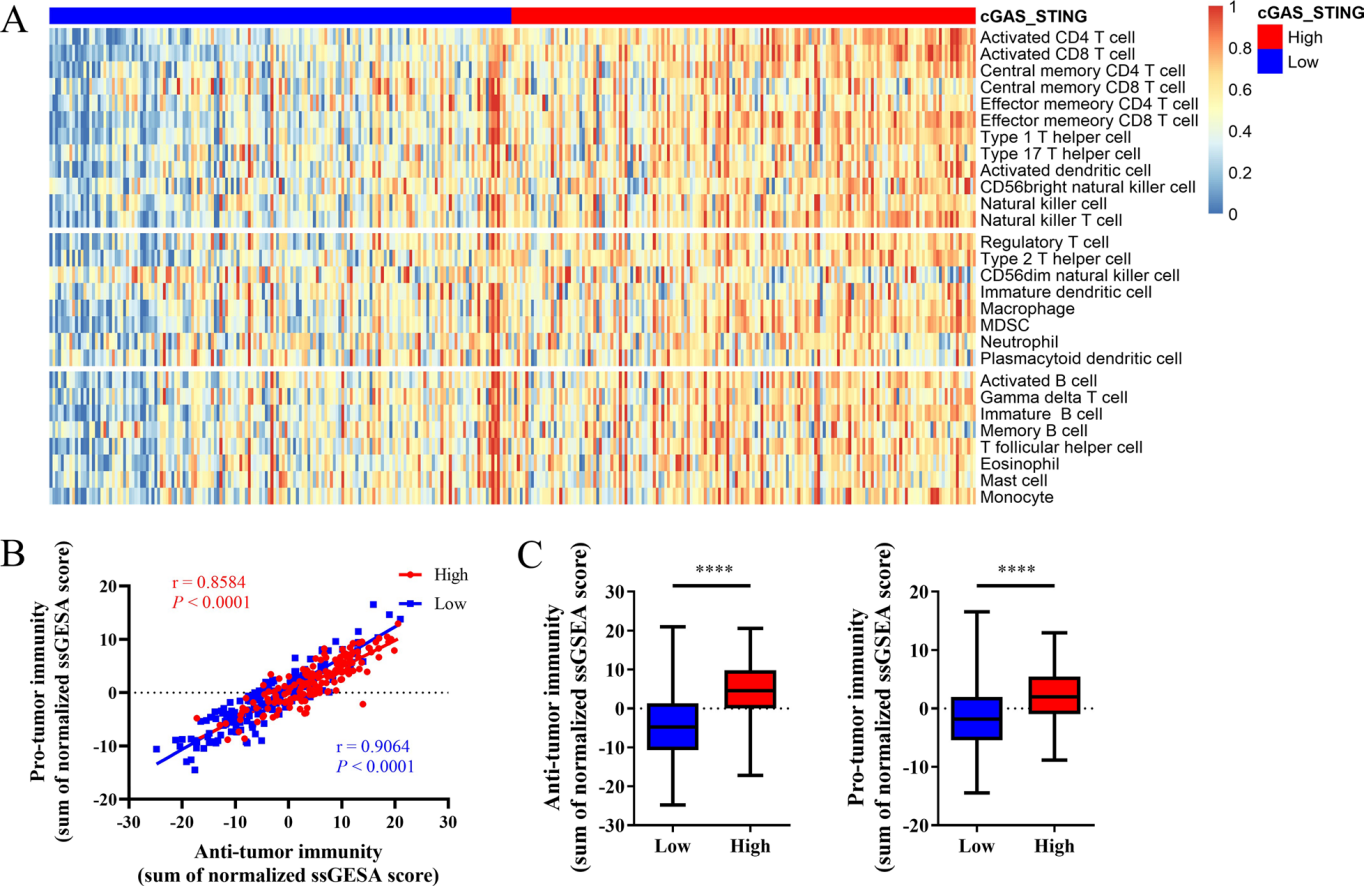
The profiles of immune cell infiltration between high and low cGAS-STING clusters. **a** The infiltration ratio of 28 immune cells. **b** Correlation of the cells with anti-tumor immunity and pro-tumor immunity. **c** Anti-tumor immunity and pro-tumor immunity score of the two cGAS-STING clusters. All r values represent Pearson correlation coefficients. Two-tailed *P* values are presented for significance (< 0.05). *P value < 0.05, **P value < 0.01, ***P value < 0.001, and ****P value < 0.0001

To evaluate the immune-related features of cGAS/STING clusters, the cases were characterized by enrichment score over the five representative signatures (Fig. 2a). The score of macrophage regulation, lymphocyte infiltration, IFN-γ response was higher in the cGAS/STING high cluster (Fig. 2b). We compared stromal fraction and leukocyte fraction between these two clusters. The results showed that higher stromal fraction and higher leukocyte fraction in the cGAS/STING high cluster (Fig. 2c). Using ESTIMATE database, we observed that higher immune score, and estimate score in the cGAS/STING high cluster (Fig. 2d). Subsequently, we compared the infiltration ratio of 28 immune cells. Positive correlations were found between cGAS/STING score and enrichment score of most types of immune cells (Additional file 1). The cGAS/STING high cluster showed relatively higher ratio of immune cell infiltration, including cells with anti-tumor activity and immunosuppressive activity (Fig. 3a). In addition, we found a positive correlation between the infiltration score of these two categories of immune cells in high and low cGAS-STING clusters (Fig. 3b). We compared the ratio of these two categories of immune cells in different cGAS/STING clusters and observed that the cGAS/STING high cluster featured both higher anti-tumor immunity and pro-tumor immunity (Fig. 3c). Positive correlations between the infiltration ratio of most types of immune cells were shown in Additional files 2–4.

### Correlation between immune-related gene signatures and cGAS/STING clusters

**Fig. 4:**
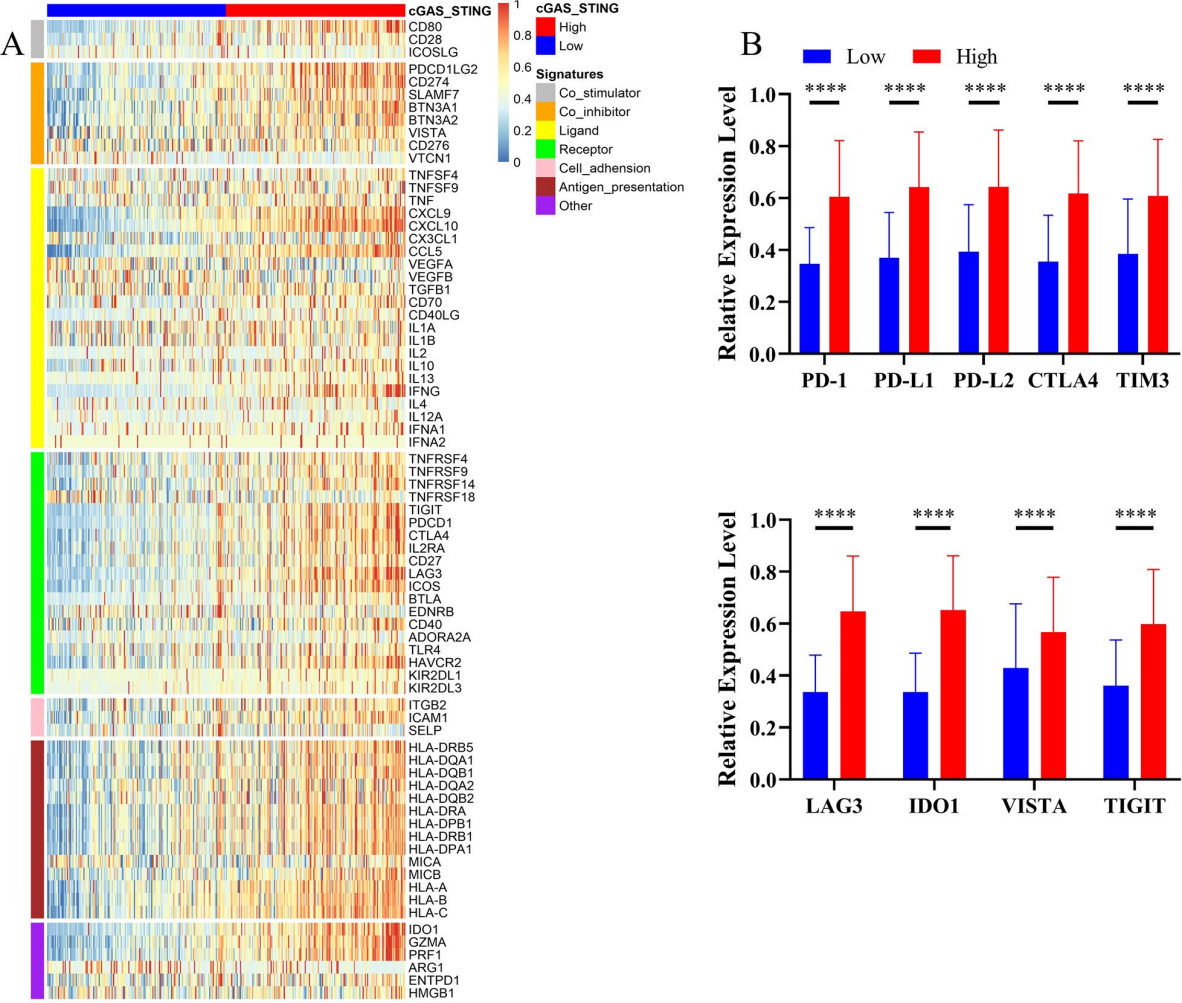
The expression profiles of 75 immune-related signatures between high and low cGAS-STING clusters. **a** Heatmap of the expression of immune signatures. **b** The expression level of immune checkpoint molecules. All *P* values for significance (< 0.05) represent comparisons via multiple t tests with FDR < 0.05. *P value < 0.05, **P value < 0.01, ***P value < 0.001, and ****P value < 0.0001

We evaluated the expression profiles of 75 immune-related genes in each cGAS/STING cluster and the cGAS/STING high cluster exhibited relatively higher expression of immune stimulatory and inhibitory signatures (Fig. 4a). We also found positive correlations between the expression of 75 immune-related genes in cGAS/STING clusters (Additional files 5–7). When comparing the expression level of several important inhibitory checkpoint molecules in cGAS/STING clusters, we found that the expression level of PD-L1, PD-L2, PD-1, CTLA4, TIM3, LAG3, IDO1, VISTA, TIGIT was higher in the cGAS-STING high cluster.

### Identification of DEGs and functional enrichment analysis

**Fig. 5:**
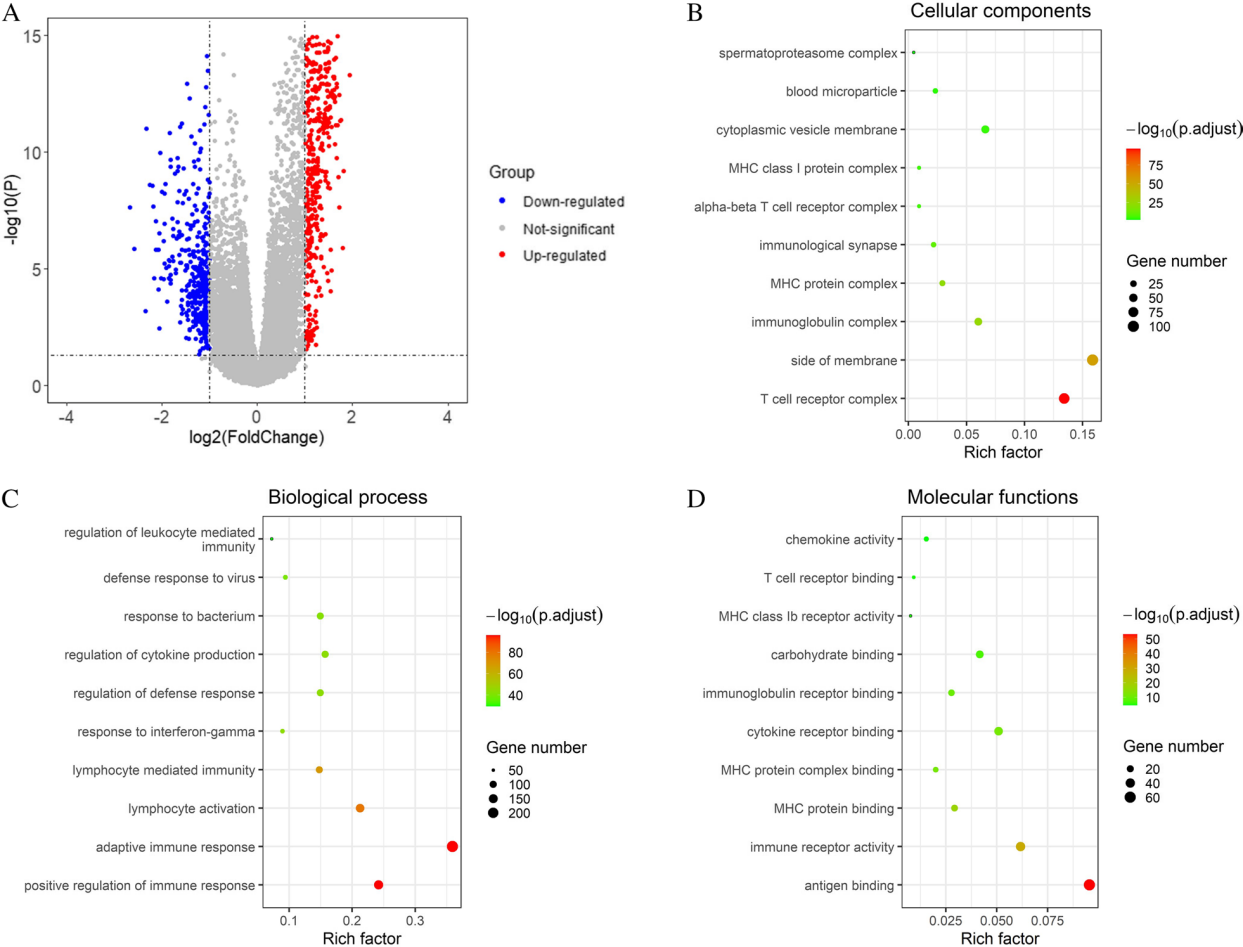
Identification of DEGs and functional enrichment analysis. **a** Volcanic diagram of DEGs based on the comparison of high/low cGAS-STING score. GO pathway enrichment analysis revealed that immune-related GO terms ranked top in molecular functions (**b**), cellular components (**c**), and biological process (**d**)

Differential expression analysis was performed between high and low cGAS-STING clusters. 676 up-regulated genes and 383 down-regulated genes were identified (Fig. 5a). We performed functional enrichment analysis of DEGs and revealed the following top immune related GO terms: T cell receptor complex, immunoglobulin complex, and MHC protein complex in cellular components (Fig. 5b); positive regulation of immune response, adaptive immune response, and lymphocyte activation in biological process (Fig. 5c); antigen binding, immune receptor activity and MHC protein binding in molecular functions (Fig. 5d).

## Discussion

In this study, we explored the correlation between the cGAS-STING pathway and the tumor microenvironment in OSCC patients. We used the expression profiles of six key molecules (cGAS, STING1, TBK1, IRF3, CCL5 and CXCL10) to represent the activation status of the cGAS-STING pathway. We found that cGAS-STING pathway is associated with OS, PFS and DFS of OSCC patients. Relatively lower cGAS-STING score was observed in T3-T4 and clinical stage III-IV groups. Downregulation of cGAS-STING signaling has been associated with poor prognosis in several tumor types [22, 23]. Accumulating data had demonstrated that activation of the cGAS-STING pathway is crucial in the TME, and the benefit of induced tumor regression and increased survival time in preclinical studies and clinical trials had been achieved by STING agonists administration [24–26]. Promising therapeutic benefit highlights the crucial role of cGAS-STING pathway in anti-tumor immunity. Several ongoing clinical trials are evaluating the potential benefit of STING agonists as monotherapies or in combination with ICI.

Immune cell infiltration has been reported as an important indicator of tumor prognosis. We firstly found higher score of macrophage regulation, lymphocyte infiltration, and IFN-gamma response in the cGAS-STING high cluster, which indicated the correlation between cGAS-STING activation and macrophages, lymphocytes in the tumor microenvironment. We analyzed the differences in infiltration ratio of 28 immune cells in high and low cGAS-STING clusters. The infiltration proportion of anti-tumor immune cells, including activated CD4^+^ T cell, activated CD8^+^ T cell and nature killer (NK) cell is higher in the cGAS-STING high cluster. We also found higher proportion of immunosuppressive cells in the high cGAS-STING cluster, such as Treg cell, macrophage and myeloid-derived suppressor cell (MDSC). These findings indicated that both anti-tumor immune cells and immunosuppressive cells are infiltrated in the tumor microenvironment when cGAS-STING pathway related gene expressions are increased. Activation of the cGAS-STING pathway has been reported not only to awake the anti-tumor response of NK cells, but also to promote DCs activation and maturation, which results in the activation and infiltration of T cells to form an inflamed TME [6–8, 27]. Studies had reported that patients with the inflamed TME have improved survival [28, 29]. Additionally, co-expression of inhibitory factors was observed after T cells infiltration [28, 30, 31]. The expressions of negative regulatory immune checkpoints, including PD-1, PD-L1, PD-L2, CTLA-4, TIM3, LAG3, IDO1, TIGIT and VISTA, were relatively higher in the cGAS-STING high cluster. The infiltration of immunosuppressive cells and elevated inhibitory pathways may be a negative feedback of anti-tumor immunity activation. Functional enrichment analysis of DEGs revealed that pathways involved in T cell immune response and antigen presentation were ranked top when the cGAS-STING pathway is activated. Besides, the efficacy of ICI treatment was abated in STING-deficient mice [32]. These indicated that patients with high cGAS-STING score may be more sensitive to ICI, the combination of these therapies may have synergistic effects. However, the limitation is that our findings are based on bioinformatics analysis, further experiments are needed to validate these findings.

## Conclusions

In summary, we investigated the correlation between cGAS-STING signaling pathway and the tumor immune microenvironment in OSCC patients. Our findings revealed potential benefit of STING agonists plus immune checkpoint inhibitors in OSCC patients.

## Supplementary Information

**Additional file 1:** Correlation between the cGAS-STING score and the enrichment score of 28 immune cells. All r values represent Pearson correlation coefficients. Two-tailed P values are presented for significance (< 0.05).

**Additional file 2:** Correlation matrix of the ratio of 28 immune cells in all cases.

**Additional file 3:** Correlation matrix of the ratio of 28 immune cells in cGAS-STING low cluster.

**Additional file 4:** Correlation matrix of the ratio of 28 immune cells in cGAS-STING high cluster.

**Additional file 5:** Correlation matrix of the expression of immune signatures in all cases.

**Additional file 6:** Correlation matrix of the expression of immune signatures in cGAS-STING low cluster.

**Additional file 7:** Correlation matrix of the expression of immune signatures in cGAS-STING high cluster.

## Data Availability

The datasets used during the current study are available from TCGA data portal (https://portal.gdc.cancer.gov/), Metascape database (http://metascape.org), and ESTIMATE database (https://bioinformatics.mdanderson.org/estimate/index.html).

## Abbreviations

cGAS: DNA-sensing receptor cyclic GMP–AMP synthase
STING: Stimulator of interferon genes
dsDNA: Double-stranded DNA
HNSCC: Head and neck squamous cell carcinoma
OSCC: Oral squamous cell carcinoma
ICI: Immune checkpoint inhibitor
TME: Tumor microenvironment
OS: Overall survival
DSS: Disease-specific survival
PFS: Progress free survival
TCGA: The cancer genome atlas
FDR: False discovery rate
ssGSEA: Single-sample gene set enrichment analysis
GO: Gene ontology
DEG: Differentially expressed gene
NK cell: Natural killer cell
MDSC: Myeloid-derived suppressor cell
DC: Dendritic cell
