# Supplementary figures and images for: Potential for treatment benefit of STING agonists plus immune checkpoint inhibitors in oral squamous cell carcinoma
Source: BMC Oral Health. 2021 Oct 8;21:506. doi: 10.1186/s12903-021-01813-8 (PMC8501566; doi:10.1186/s12903-021-01813-8)

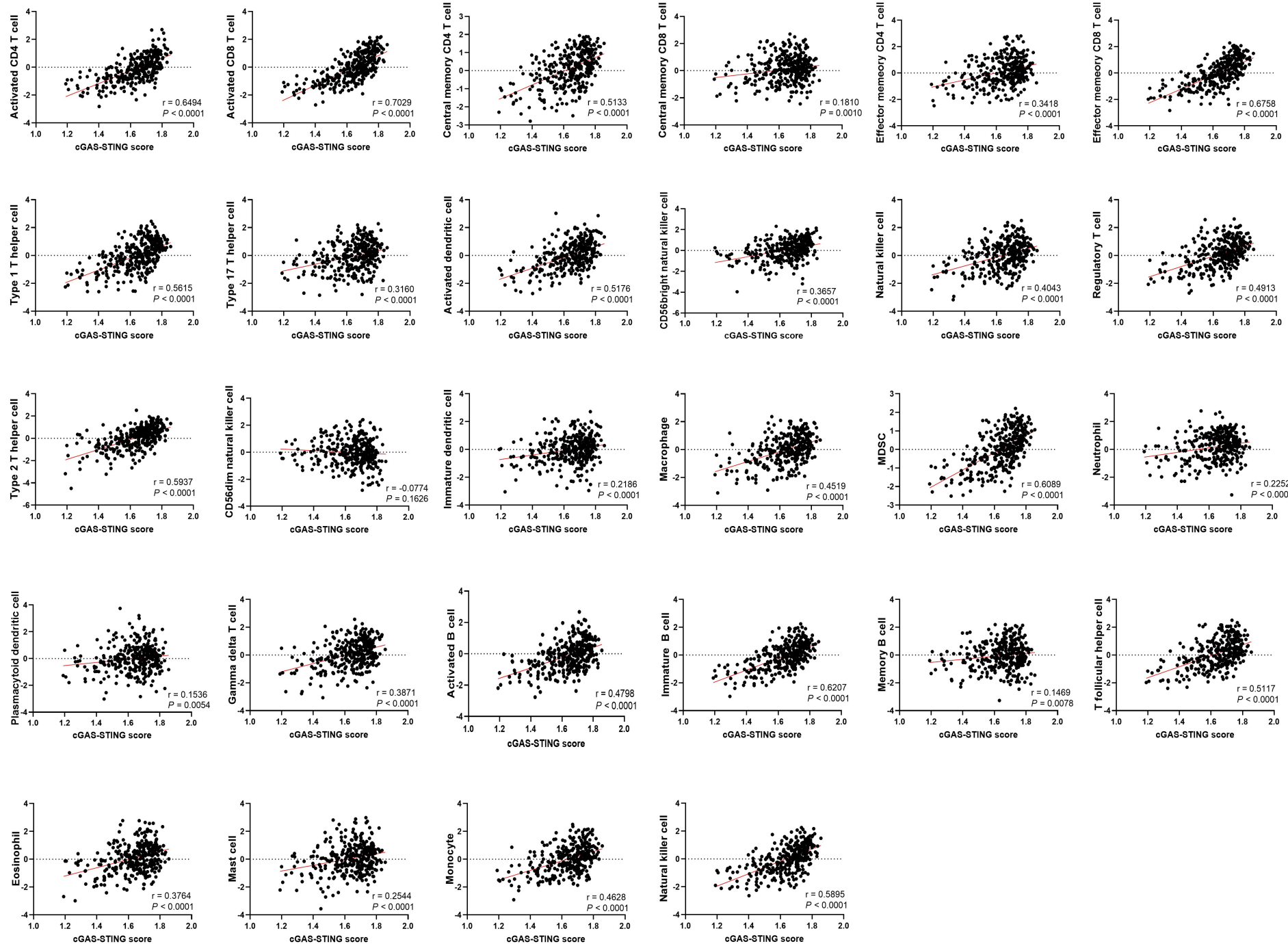

Supplement: Supplementary file 1 — Additional file 1: Correlation between the cGAS-STING score and the enrichment score of 28 immune cells. All r values represent Pearson correlation coefficients. Two-tailed P values are presented for significance (< 0.05). [file 12903_2021_1813_MOESM1_ESM.pdf]

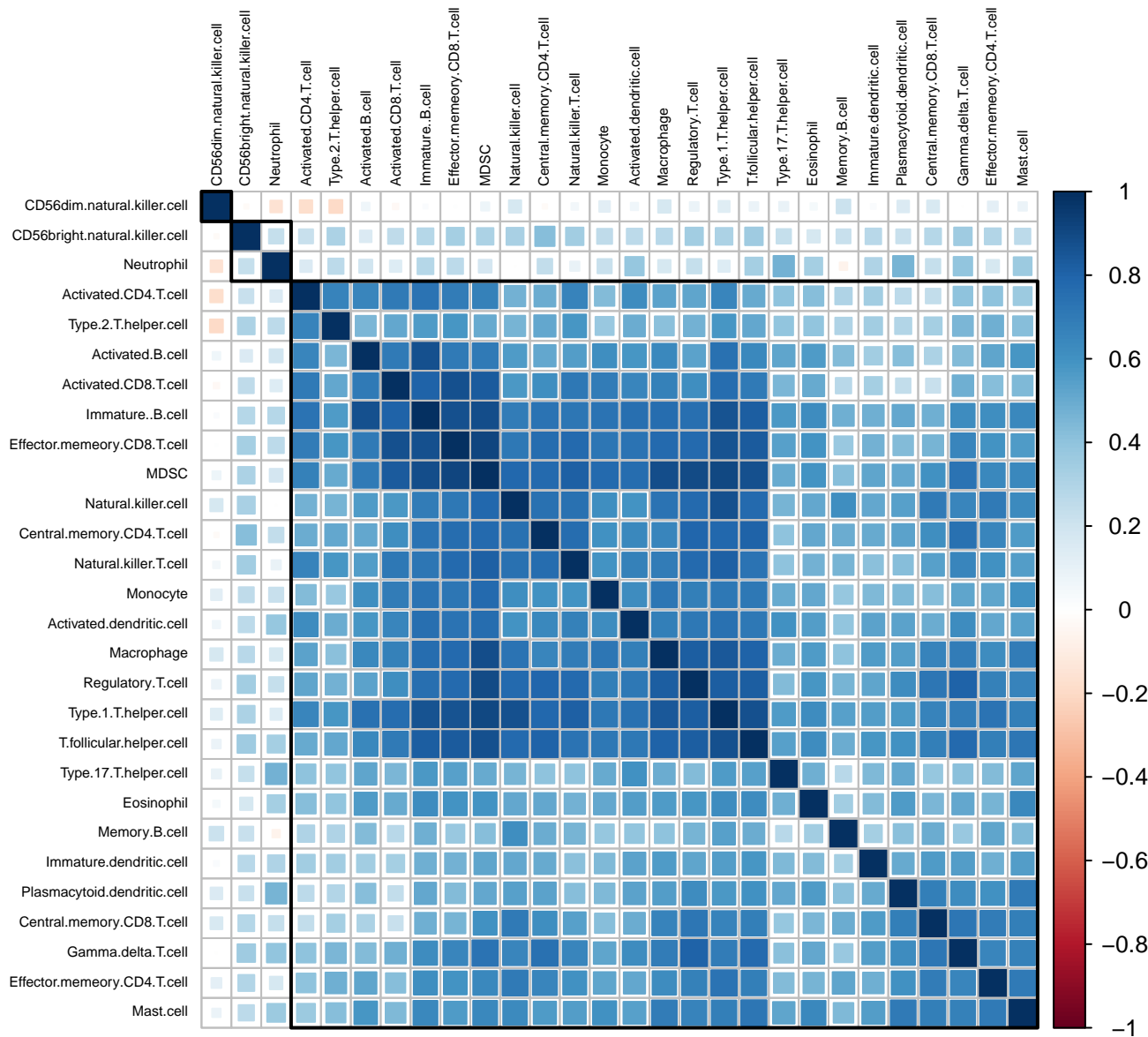

Supplement: Supplementary file 2 — Additional file 2: Correlation matrix of the ratio of 28 immune cells in all cases. [file 12903_2021_1813_MOESM2_ESM.pdf]

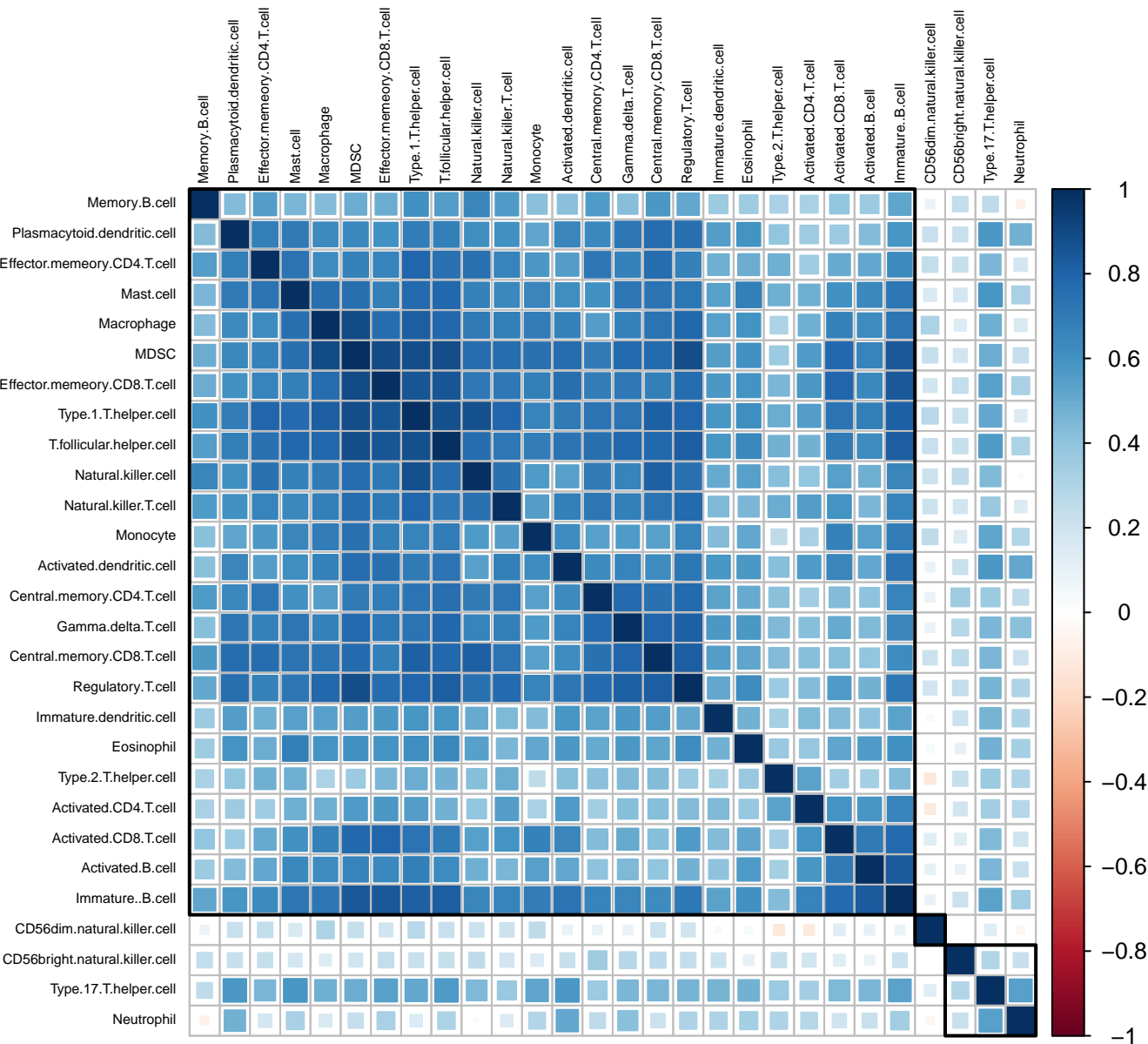

Supplement: Supplementary file 3 — Additional file 3: Correlation matrix of the ratio of 28 immune cells in cGAS-STING low cluster. [file 12903_2021_1813_MOESM3_ESM.pdf]

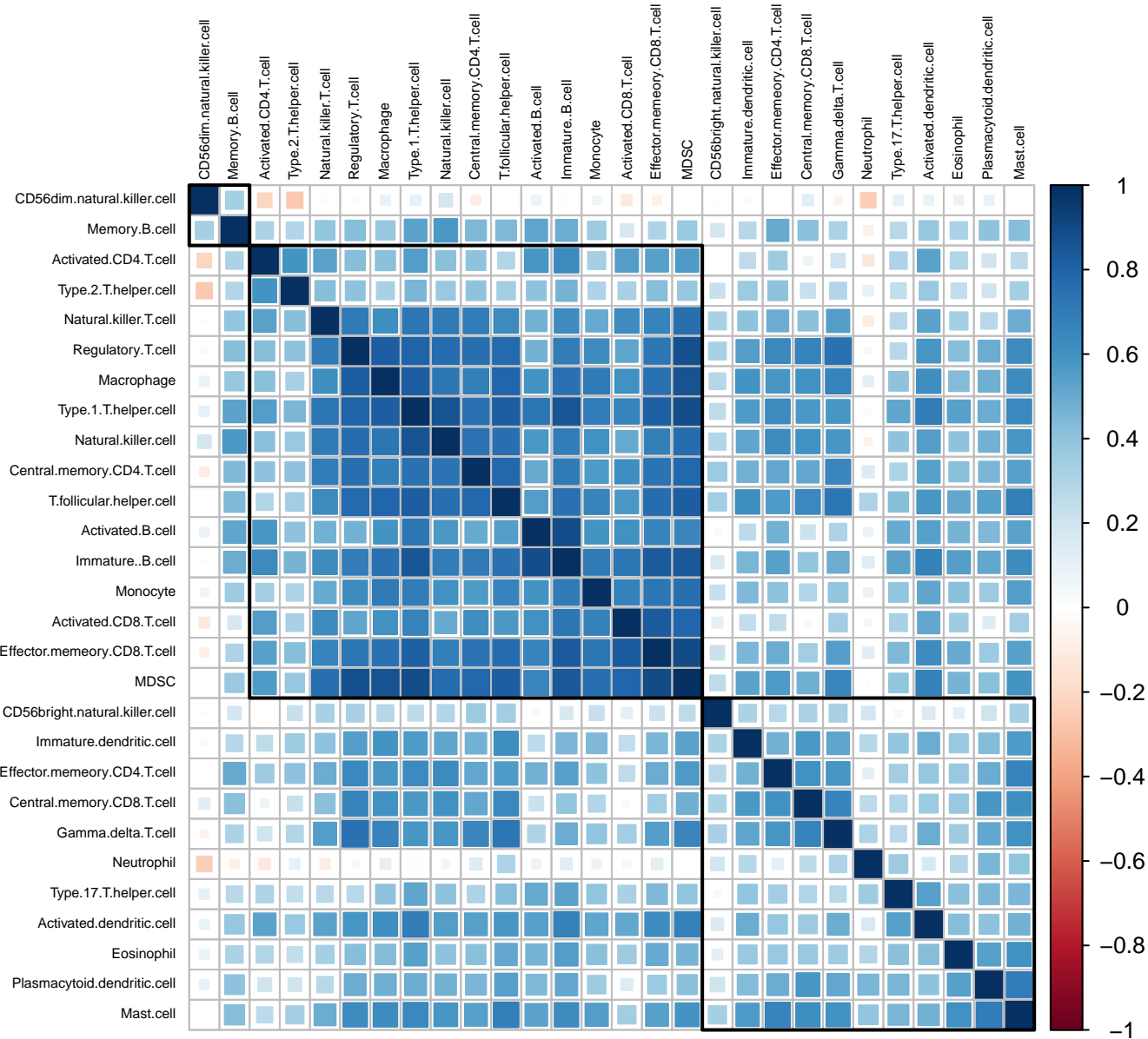

Supplement: Supplementary file 4 — Additional file 4: Correlation matrix of the ratio of 28 immune cells in cGAS-STING high cluster. [file 12903_2021_1813_MOESM4_ESM.pdf]

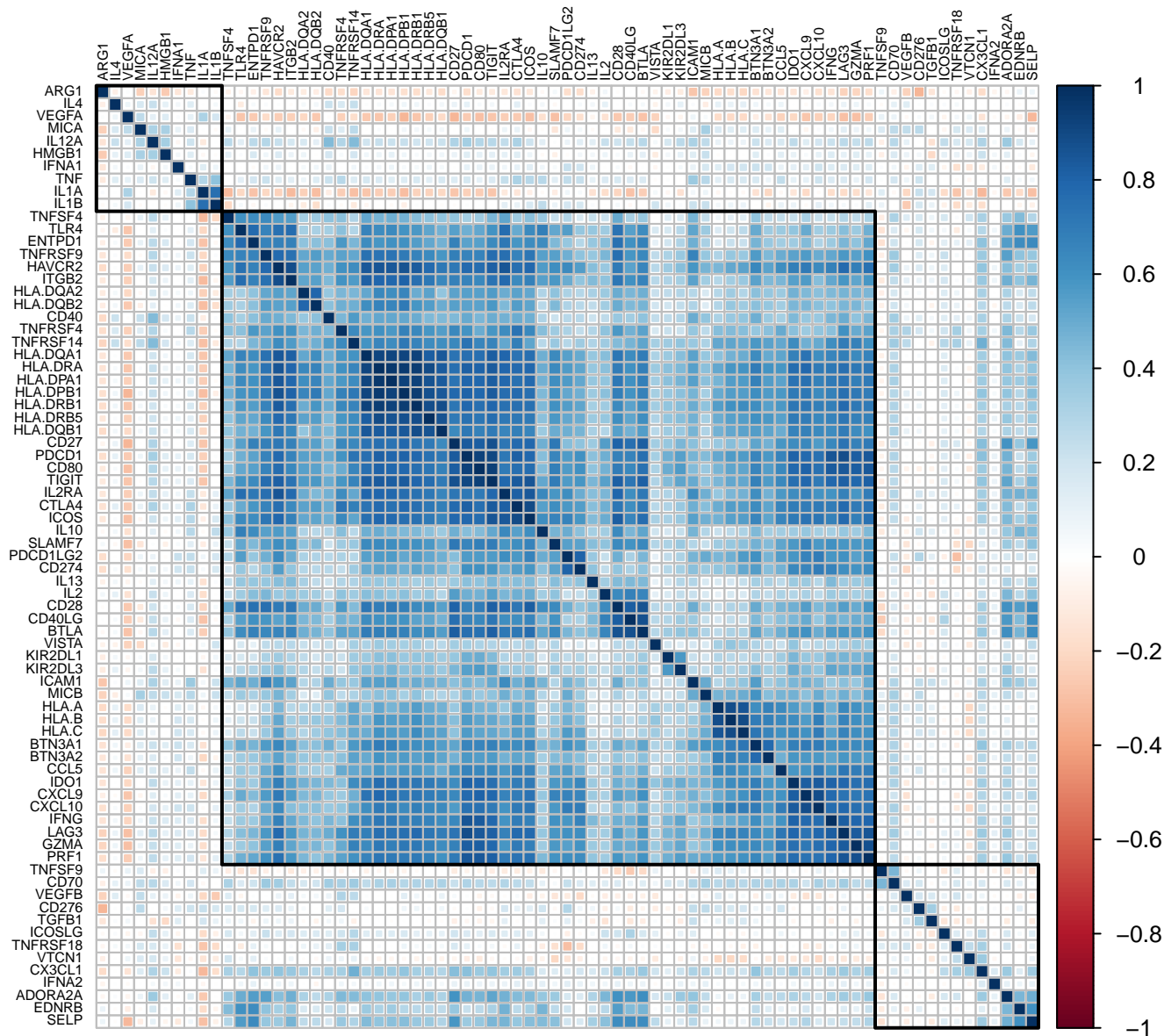

Supplement: Supplementary file 5 — Additional file 5: Correlation matrix of the expression of immune signatures in all cases. [file 12903_2021_1813_MOESM5_ESM.pdf]

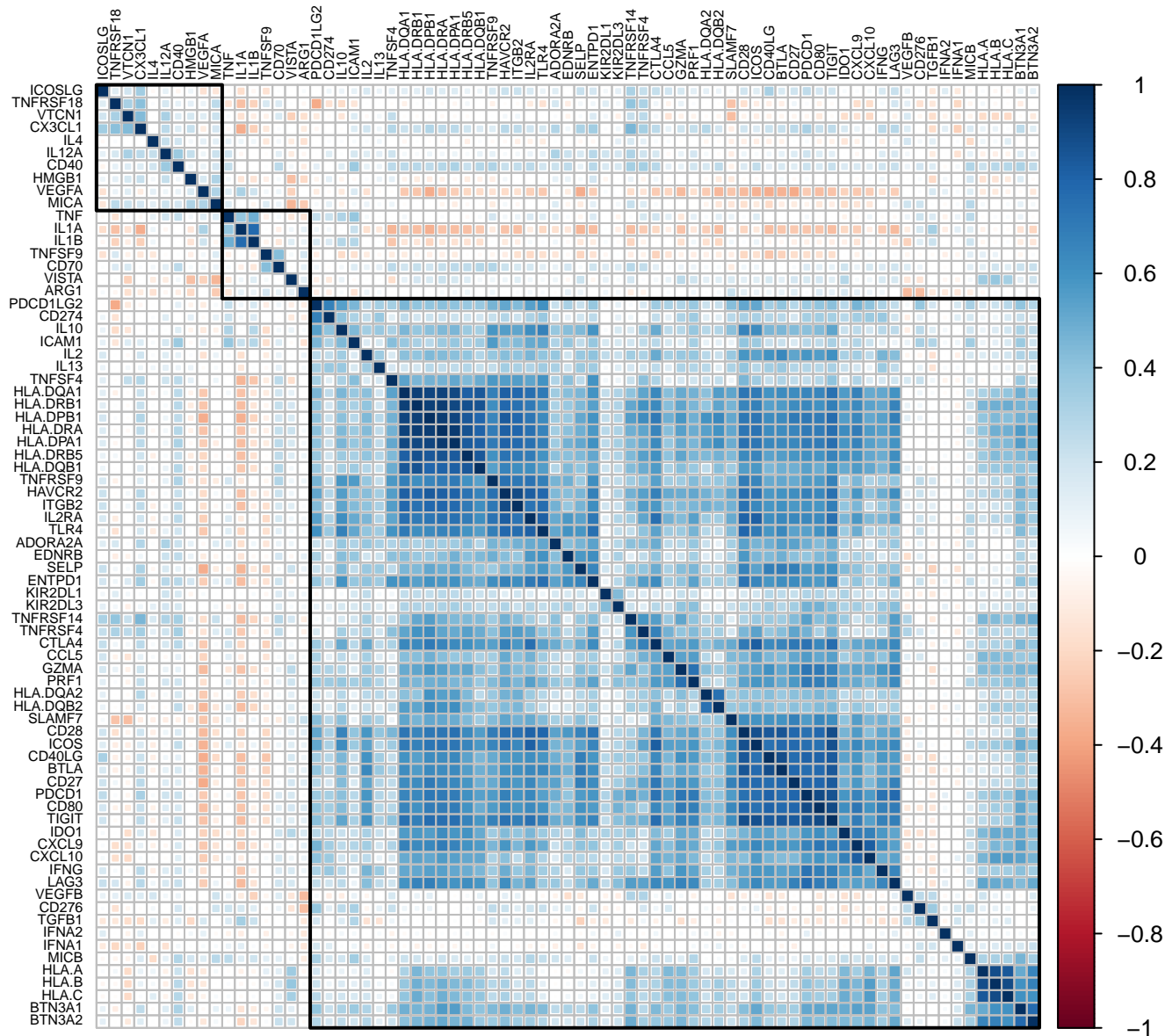

Supplement: Supplementary file 6 — Additional file 6: Correlation matrix of the expression of immune signatures in cGAS-STING low cluster. [file 12903_2021_1813_MOESM6_ESM.pdf]

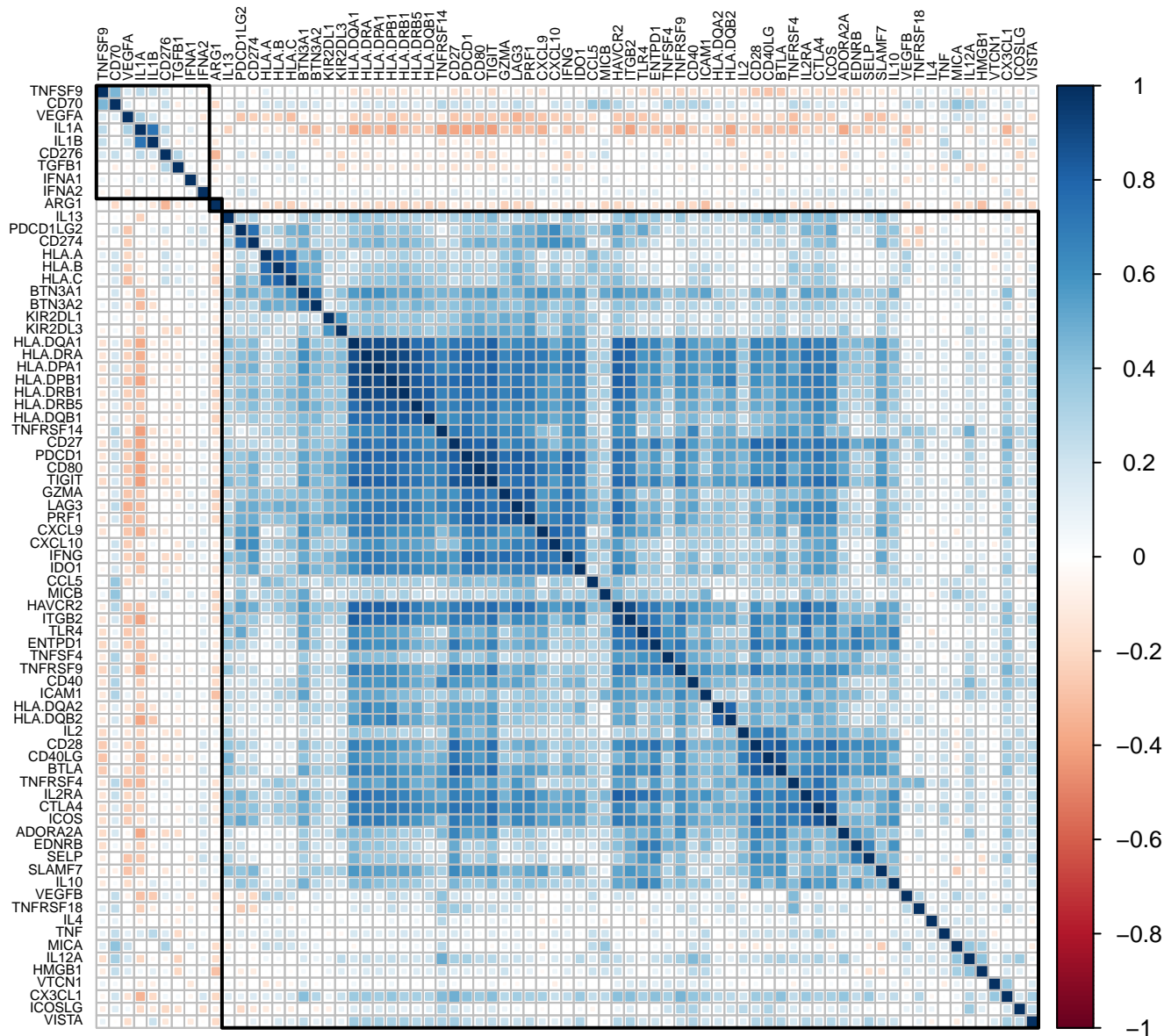

Supplement: Supplementary file 7 — Additional file 7: Correlation matrix of the expression of immune signatures in cGAS-STING high cluster. [file 12903_2021_1813_MOESM7_ESM.pdf]
